# Molecular and Clinical Characterization of Chikungunya Virus Infections in Southeast Mexico

**DOI:** 10.3390/v10050248

**Published:** 2018-05-09

**Authors:** Kame A. Galán-Huerta, Erik Martínez-Landeros, Juan L. Delgado-Gallegos, Sandra Caballero-Sosa, Iliana R. Malo-García, Ildefonso Fernández-Salas, Javier Ramos-Jiménez, Ana M. Rivas-Estilla

**Affiliations:** 1 Departamento de Bioquímica y Medicina Molecular, Facultad de Medicina, Universidad Autónoma de Nuevo Leon, Av. Francisco I. Madero S/N, Mitras Centro, Monterrey, 64460 Nuevo Leon, Mexico; kame.galanhr@uanl.edu.mx (K.A.G.-H.); juan_luisdg@outlook.com (J.L.D.-G.); 2 Hospital Metropolitano Dr. Bernardo Sepulveda, Secretaría de Salud de Nuevo Leon, Adolfo Lopez Mateos No. 4600, Bosques del Nogalar, San Nicolas de los Garza, 66480 Nuevo Leon, Mexico; dr_erikmtz@itesm.mx; 3 Clínica Hospital Dr. Roberto Nettel Flores, Instituto de Seguridad y Servicios Sociales de los Trabajadores del Estado, Av. Tuxtepec y Oaxaca S/N, Francisco Villa, Tapachula, 30740 Chiapas, Mexico; sandyluzcs@hotmail.com; 4 Centro Regional de Investigación en Salud Publica, Instituto Nacional de Salud Publica 4a Avenida Norte, esquina con calle 19 poniente S/N, Centro, Tapachula, 30700 Chiapas, Mexico; malo@insp.mx (I.R.M.-G.); ildefonso.fernandezsl@uanl.edu.mx (I.F.-S.); 5 Facultad de Ciencias Biologicas, Universidad Autonoma de Nuevo Leon, Av. Pedro de Alba S/N, Ciudad Universitaria, San Nicolas de los Garza, 66455 Nuevo Leon, Mexico; 6 Servicio de Infectologia—Hospital Universitario Dr. Jose Eleuterio Gonzalez, Facultad de Medicina, Universidad Autonoma de Nuevo Leon, Av. Francisco I. Madero and Eduardo Aguirre Pequeño S/N, Mitras Centro, Monterrey, 64460 Nuevo Leon, Mexico; javramos31@gmail.com

**Keywords:** alphavirus, Chikungunya fever, phylogeny, Mexico

## Abstract

Chikungunya fever is an arthropod-borne infection caused by Chikungunya virus (CHIKV). Even though clinical features of Chikungunya fever in the Mexican population have been described before, there is no detailed information. The aim of this study was to perform a full description of the clinical features in confirmed Chikungunya-infected patients and describe the molecular epidemiology of CHIKV. We evaluated febrile patients who sought medical assistance in Tapachula, Chiapas, Mexico, from June through July 2015. Infection was confirmed with molecular and serological methods. Viruses were isolated and the *E1* gene was sequenced. Phylogeny reconstruction was inferred using maximum-likelihood and maximum clade credibility approaches. We studied 52 patients with confirmed CHIKV infection. They were more likely to have wrist, metacarpophalangeal, and knee arthralgia. Two combinations of clinical features were obtained to differentiate between Chikungunya fever and acute undifferentiated febrile illness. We obtained 10 CHIKV *E1* sequences that grouped with the Asian lineage. Seven strains diverged from the formerly reported. Patients infected with the divergent CHIKV strains showed a broader spectrum of clinical manifestations. We defined the complete clinical features of Chikungunya fever in patients from Southeastern Mexico. Our results demonstrate co-circulation of different CHIKV strains in the state of Chiapas.

## 1. Introduction

Chikungunya virus (CHIKV) is transmitted by the bite of the *Aedes aegypti* and *Aedes albopictus* mosquitoes. It produces an acute illness with high fever, joint pain, head, and muscular pain. Even though it rarely causes death, joint pain can last months or years, develop chronicity and cause disability [1,2]. There are neither specific antiviral drugs nor available vaccines to prevent the infection.

Since its discovery in 1952, CHIKV has caused massive outbreaks in Africa and Southeast Asia [3,4]. One of the most studied CHIKV outbreaks was the one that occurred in La Réunion Island in 2005, where clinical manifestations were recorded in detail and chronic pain was reported [5,6,7,8]. From December 2013 through the epidemiological week 45 in 2017, CHIKV has caused more than 331,000 confirmed infections in the American continent [9]. In Mexico, until 4 November 2017, there have been more than 12,500 confirmed cases [10].

Chikungunya virus is a member of the *Alphavirus* genus in the *Togaviridae* family and its genome is approximately 12 kb in length [11]. There are four identified CHIKV lineages: West African, East/Central/South African (ECSA), Asian, and Indian Ocean lineage. The later was derived from the ECSA lineage and arose from La Réunion outbreak. This new lineage had the substitution A226V at the E1 protein, which together with other mutations, allowed the infection of *A. albopictus* mosquitoes in a greater extent [12,13]. The Asian lineage is the responsible for the outbreak in the American continent [1,14,15,16]. Nevertheless, the ECSA lineage also circulates in Brazil [17,18,19].

Chikungunya fever (CHIKF) is characterized by an abrupt febrile illness, polyarthralgia and maculopapular rash, after 2 to 4 days of incubation period. Arthralgia can be incapacitating and last for weeks. In contrast to other illnesses, the majority of the infections (85%) are symptomatic [6,20]. Arthralgia is symmetrical and bilateral, localized at the upper and lower limbs [21]. According to studies conducted during La Réunion outbreak the affected joints were: ankles, knees, hands, wrists, feet, shoulder and elbows. Fifty-four of the patients reported rash, particularly on trunk and arms. Arthritis we reported by 45% of the patients, being the ankle was the most affected joint. Myalgia and headache affected more than half of the patients [5,8,22,23]. The case definition of acute clinical cases includes fever and joint pain with acute onset and/or epidemiological and laboratory criteria. Epidemiological criteria consist of residing or traveling to an endemic CHIKV region. A chronic case consists of previous clinical diagnosis of CHIKF 12 weeks after symptom onset and presentation with at least one joint manifestation that is continuous or recurrent [24,25].

Even though clinical features of CHIKF in Mexican population have been described before [26,27,28], arthralgia, arthritis, and rash are mentioned as a whole without describing the specific affected joints or areas. A detailed analysis of the spectrum of clinical signs can help to identify specific manifestations that could aid clinicians to distinguish CHIKF from other similar viral illnesses. Although there are a great number of available CHIKV sequences, Mexican sequences are scarce. In addition, the existence of different CHIKV circulating in Chiapas and if there are associated clinical features is unknown. The aim of this study was to make a full description of the clinical features in Chikungunya-infected patients, and the molecular epidemiology of CHIKV.

## 2. Materials and Methods

### 2.1. Ethical Aspects

The study was conducted in accordance with the Declaration of Helsinki, and the protocol was reviewed and approved by the Ethics Committee from Facultad de Medicina y Hospital Universitario—Universidad Autónoma de Nuevo León with the following registration number: IF12-003 (approval date: 20 August 2012). All study participants provided informed written consent prior to study enrollment. All patient samples and data were assigned institution-specific identification numbers to ensure patient anonymity.

### 2.2. Study Population, Recruitment and Sample Collection

We studied patients who sought medical assistance at the secondary-level Hospital Clinic “Dr. Roberto Nettel Flores” in Tapachula, Chiapas, Mexico, from June through July 2015. This health clinic receives only State workers and their immediate relatives, not open to the general population. Patients were eligible for enrollment if they were ≥18 years old and febrile (temperature ≥37.5 °C) or had history of fever within last five days, accompanied by one or more of the following symptoms: polyarthralgia, headache and/or rash. Individuals with another identifiable infection were excluded. Clinical information and physical examination were recorded. The visual analogue scale (VAS) was used to evaluate pain intensity and patients were inquired if they needed help to fulfill simple tasks. A single whole blood sample was collected at the enrollment day and sera samples were stored at −70 °C until further use. Fourteen months after the onset of symptoms, patients were contacted by telephone and inquired for CHIKF symptomatology. Patient home addresses were used to identify longitude and latitude coordinates. ArcGIS v10.2.2 (ESRI, Redlands, CA, USA) was used to overlay the resulting points and carry out the geospatial analysis. According to the female mosquito flight range, a 400 m buffer distance was applied to each positive dwelling [29].

### 2.3. Viral RNA Detection

RNA extraction was performed using QIAamp UltraSens Virus Kit (QIAGEN, Valencia, CA, USA). Screening for CHIKV was carried out using the nsp4 one-step, probe-based, real-time reverse transcription PCR assay (rRT-PCR) described by Lanciotti [30]. Chikungunya positive and negative samples were screened and serotyped for Dengue viruses (DENV) using the protocol described by Seah [31]. Chikungunya negative samples were screened for Zika virus (ZIKV) using the rRT-PCR assay with the primer/probe set with higher sensitivity described by Lanciotti [32]. All rRT-PCR assays were done using the SuperScript III Platinum One-Step qRT-PCR kit (Invitrogen, Carlsbad, CA) in a 7500 Fast Real-Time PCR System (Applied Biosystems, Carlsbad, CA, USA).

### 2.4. Specific IgM Enzyme-Linked Immunosorbent Assay (ELISA)

InBios CHIKjj Detect IgM ELISA (Seattle, WA, USA) was used to screen sera for the presence of CHIKV-specific IgM antibodies, following manufacturer’s instructions [33]. Samples were designated as positive for CHIKV-specific IgM if they returned a value of ≥1 and as negative at <1. We used an in-house, luminometric, E protein-based ELISA to detect dengue-specific IgM antibodies (R. Vidaltamayo, unpublished data [34]). The ELISA plates were read at the GloMax Discover System (Promega, Madison, WI, USA). All samples were run in triplicate.

### 2.5. E1 Gene Amplification and Sequencing

The PrimeScript RT-PCR Kit (TaKaRa, Shiga, Japan) was used to generate one PCR amplicon (1623 nt) from the E1 region. The amplicon was then purified using the QIAquick PCR Purification Kit (Qiagen). Sequencing was performed on a 3500 Genetic Analyzer (Applied Biosystems) using BigDye Terminator v3.1 cycle sequencing kit (Applied Biosystems). Sequences were assembled based on the reference Chikungunya virus sequence from NCBI: NC_004162.2. The sequences were deposited in GenBank under the following accession numbers: MG525024—MG525033.

### 2.6. Phylogenetic Analysis

The newly obtained *E1* gene nucleotide sequences and sequences from GenBank were aligned using the MUSCLE algorithm. We included sequences of the four previously described CHIKV lineages (West Africa, ECSA, IOL and Asian). The final data set comprised of 73 E1 coding sequences (1317 nt in length) from 32 countries isolated during 1953–2015 (Table S1). The evolutionary history was inferred using the maximum-likelihood method in MEGA v6.06 (Arizona State University, Tempe, AZ, USA) [35] and the maximum clade credibility (MCC) in BEAST v1.8.2 (University of Edinburgh, Edinburgh, United Kingdom) [36]. For the phylogenic reconstruction, we used the Hasegawa-Kishino-Yano substitution model [37] with a discrete Gamma distribution (4 categories). For the MCC approach we used a strict clock model with a coalescent constant size tree. In the maximum-likelihood approach we used a bootstrap of 1000 replicates and in the maximum clade credibility approach, 10 million generations with 10% removed as burn-in. The trace files generated were visualized in the software package Tracer v1.6 (University of Edinburgh) [38]. The obtained effective sample size was greater than 200. The best-supported tree was obtained with the TreeAnotator program excluding the first 10% of trees as burn-in.

### 2.7. Statistical Analysis

The frequencies of patient demographic and clinical characteristics were determined. The outcome for analysis was having a confirmed infection with CHIKV, defined as a positive result on CHIKV rRT-PCR or positive CHIKV-specific IgM antibodies. Bivariable associations with a confirmed CHIKV infection were determined using the Fisher’s exact test. Continuous variables with normal distribution were analyzed with Student’s *t*-test and variables with non-normal distribution with Mann-Whitney U test. All variables with a *p*-value less than or equal to 0.25 were considered in the multivariable model, which was determined using binary logistic regression using a backward stepwise selection procedure (entry: 5%, exit: 10%). Odds ratios were adjusted for age and gender. Data was entered and analyzed using IBM SPSS Statistics v20 (IBM Corporation, Armonk, NY, USA) for analysis. All *p*-values <0.05 were considered significant. Positive predictive values (PPVs), negative predictive values (NPVs), sensitivity, specificity and likelihood ratios of different combinations of clinical features were used to distinguish CHIKF from other acute undifferentiated fever illness (AUFI).

## 3. Results

There were 61 patients who met the inclusion criteria and agreed to participate in the study. Sixty seven percent were female and the median age was 43 years old, ranging from 18 to 69 years. Eleven percent reported previous dengue fever (confirmed by serology) and 39% reported that others in their household had been febrile in a two-week interval prior to the interview.

From the 61 enrolled patients, 52 (85%) were positive for CHIKV infection (CHIKV+) either by rRT-PCR or IgM ELISA. Among the positive cases, 33 patients (64%) had CHIKV viral RNA detected in serum by rRT-PCR and 19 patients (36%) had IgM anti-CHIKV antibodies. No samples were positive for the two diagnostic tests. Samples were positive by rRT-PCR from day one to four after onset of symptoms. IgM anti-CHIKV antibodies were detected from day two to day nine. The remaining nine patients (CHIKV negative) were evaluated for DENV and ZIKV by RT-PCR and rRT-PCR, respectively. We found neither DENV nor ZIKV viral RNA nor IgM anti-DENV antibodies in the serum samples. Therefore, these patients were classified as AUFI. Because DENV is endemic in the Chiapas state, DENV co-infection was investigated. Chikungunya rRT-PCR positive samples were evaluated for DENV by RT-PCR. However, we found no CHIKV and DENV co-infection in the studied patients. As for ZIKV, at the time of patient enrollment, there was no ZIKV circulation at the Chiapas state. Nevertheless, ZIKV was searched intentionally in the negative samples.

**Figure 1:**
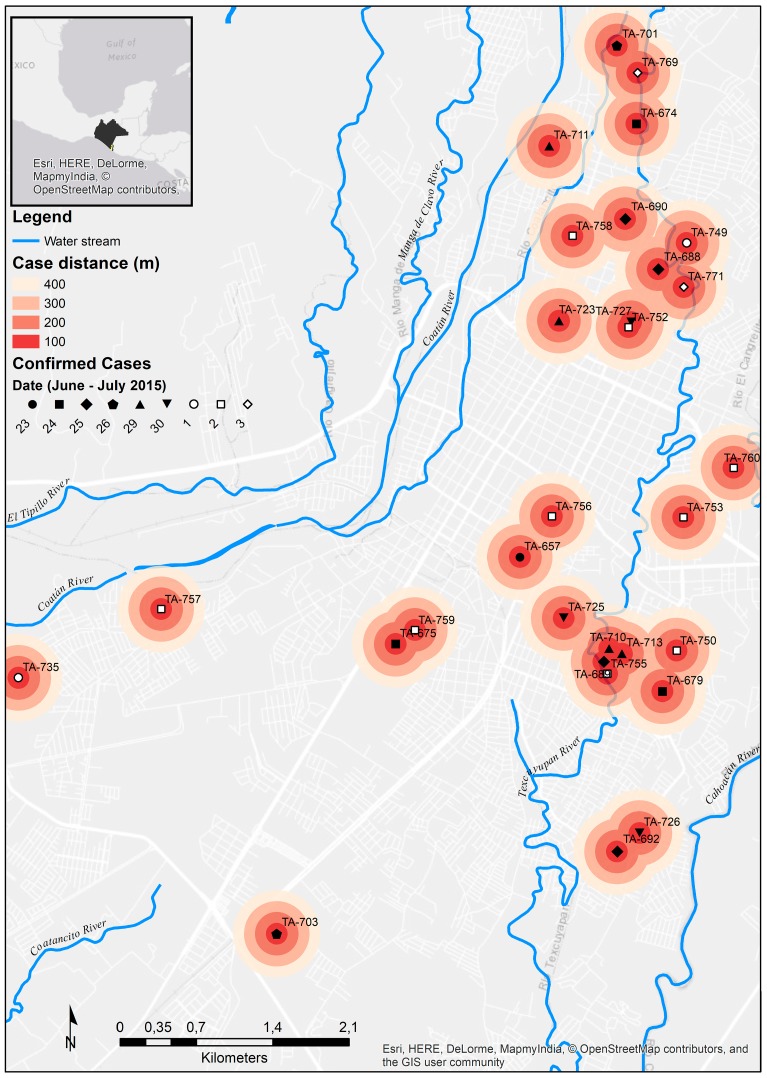
Distribution of confirmed Chikungunya fever cases in Tapachula, Chiapas. Chikungunya fever cases are marked. Concentric distance in meters is shown. The date of the patient’s enrolment is indicated. Patients enrolled in June and July are marked with a filled and hollow shape, respectively. The upper left square indicates the location of Tapachula municipality and Chiapas state. We used the World Light Gray Base by Esri (Esri Inc., Redlands, CA, USA) [41].

The CHIKV+ patients were distributed across eight Chiapas’ municipalities. Thirty-nine patients (75%) lived in the city of Tapachula. We were able to obtain 39 home addresses of the confirmed CHIKV cases, 30 from Tapachula and nine from the rest of the cities. Patients from Tapachula had their dwellings distributed across the city. Based on well-documented average flight range distance of less than 400 m for *A. aegypti* females, a 200 m range was applied to the patient’s dwelling [39,40]. This resulted with seven clusters of cases across the city. Five clusters involved two dwellings and two clusters involved three to four dwellings (Figure 1). The mean distance between each dwelling in the clusters was 240 m.

### 3.1. Comparison of Chikungunya—Positive Patients versus Acute Undifferentiated Febrile Illness Patients

When we compared CHIKV+ patients with AUFI patients, there was no difference in the time of onset of symptoms (both median 3 d). Patients with confirmed CHIKF were older than AUFI patients (mean years 42.8 and 36.4, respectively), but not significant. Chikungunya-infected patients were predominantly females (73%) compared to AUFI patients that were predominantly males (67%), being significant (*p* = 0.04).

There were no significant differences in clinical presentations between those with CHIKV confirmed infection and those without (Table 1). However, CHIKV+ patients reported arthritis and adenopathy in a greater proportion. Arthritis distribution is shown in Table 2. One patient, chikungunya-infected, had five joints affected by arthritis. Retroauricular adenopathy was the most commonly reported presentation.

**Figure 2:**
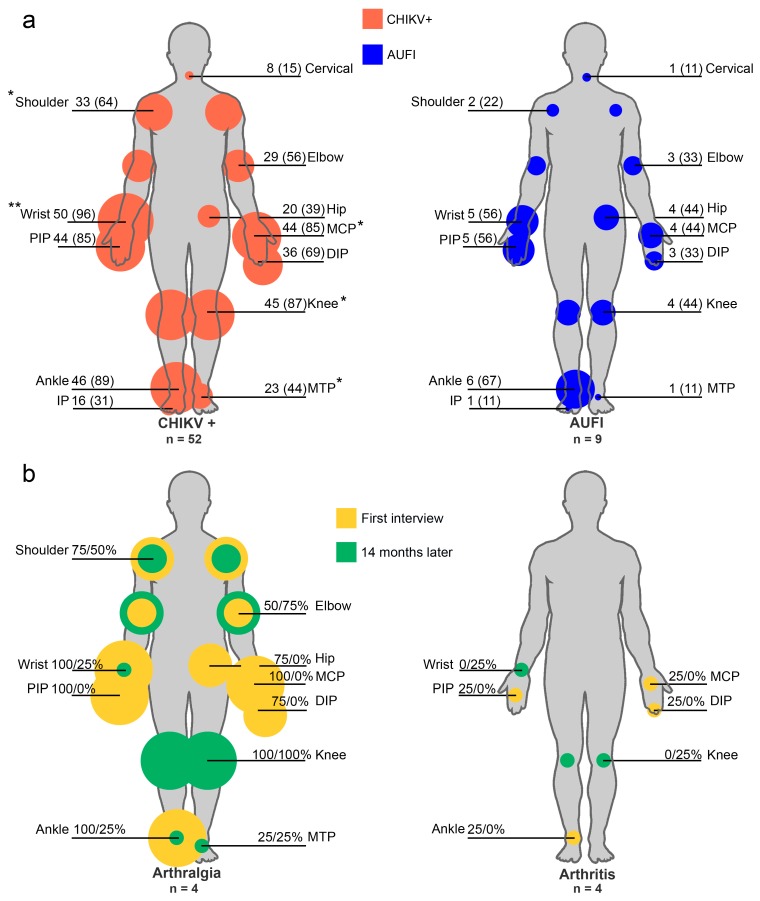
Arthralgia and arthritis distribution in the studied patients from Tapachula, Chiapas, June through July 2015. (**a**) Arthralgia distribution in the CHIKV+ and AUFI patients. The reported arthralgia was symmetrical. For illustrative purposes only, unilateral joints are indicated. Values are no. (%) patients; (**b**) Arthralgia and arthritis distribution on the first interview and after 14 months in the four studied patients. Values are % of patients in the first and second interview. CHIKV+, positive for chikungunya virus infection; AUFI, acute undifferentiated febrile illness; MCP, metacarpophalangeal joint; PIP, proximal interphalangeal joint; DIP, distal interphalangeal joint; MTP, metatarsophalangeal joint. * *p* < 0.05, ** *p* < 0.01, Fisher exact test.

Patients manifested symmetrical arthralgia. Chikungunya positive patients reported a higher number of arthralgia-involved joints (median 38) compared to AUFI patients (median 13) being significant (*p* < 0.01). In CHIKV+ patients, arthralgia was present in a significantly greater proportion in specific joints (Figure 2a). Rash was located mainly in upper limbs, leaving hands unaffected. Lower limbs were also affected, especially in CHIKV+ patients (Table 2).

Forty-nine percent of the patients reported to need some help to fulfill their daily activities (*n* = 45), and all of them were CHIKV+ patients. Patients needed help mostly for lifting from a sitting position and walking (74 and 68% respectively). Chikungunya-infected patients had higher VAS score (median 8) compared to the AUFI patients (median 7) (*p* < 0.01). The chikungunya-infected female patients reported more pain (median 9) than males (median 7) (*p* < 0.01). Pain was not associated with the increasing age.

After the multivariate analysis, patients who had confirmed CHIKV infection were significantly more likely to have wrist, metacarpophalangeal and knee arthralgia. Similarly, patients who had confirmed CHIKV infection were significantly more likely to have a high VAS score and a high number of arthralgia involved joints (Table 3).

We could interview four CHIKV+ patients, out of 52, after 14 months of the onset of symptoms. The mean age was 51 years old and 75% were female. None of them considered themselves cured after the 14 months. Three patients reported a pain-free period. When interviewed, all the patients still had pain. The male patient reported having higher pain at the second interview (VAS score 4 vs. 7). Half of the patients reported that pain negatively affected their quality of life, and three-fourths reported mood change. Half of the patients still required some help to fulfill their daily activities. The arthralgia and arthritis distribution presented by the four studied patients at enrollment day and after 14 months is shown in Figure 2b. Hand arthralgia resolved in almost all patients, in contrast, knee arthralgia continued in all of them. Half of the patients developed arthralgia in previously pain-free joints. Hand arthritis resolved in the affected patients. Nevertheless, two patients developed knee and wrist arthralgia (Figure 2b).

### 3.2. Comparison of Patients Diagnosed by rRT-PCR versus IgM ELISA

We compared patients that were CHIKV+ by rRT-PCR and IgM ELISA. Overall, there were almost no differences. The proportion of female patients confirmed by rRT-PCR and ELISA was 61% and 95% respectively. Ninety-three percent of the total males were confirmed as CHIKV+ by rRT-PCR. Patients confirmed by rRT-PCR and ELISA showed the same clinical manifestations (Table 1). The exception was adenopathy, which was present at a greater proportion in ELISA-confirmed patients but not significant. Patients from both groups showed similar arthralgia distribution. The elbow was affected on a greater proportion in patients confirmed by rRT-PCR (*p* = 0.04). Rash affected a greater proportion of patients in the IgM group (Table 2). The number of rash affected areas increased as the time post-onset of symptoms increased. Sixty percent of the patients in the rRT-PCR group required help to fulfill their daily activities, compared to the IgM group where only 28% required help (*p* > 0.05). Patients from rRT-PCR and IgM group had similar VAS scores, median of 9 and 8 respectively. Females from the RT-qPCR group reported greater pain than males (*p* = 0.001).

### 3.3. Distinguishing Chikungunya Fever from Acute Undifferentiated Febrile Illness Using Clinical Features

We found two combinations of clinical features that can be used to differentiate CHIKF from AUFI. One option considers arthralgia in wrist, metacarpophalangeal joints, and knee. The other one considers arthralgia in the same areas plus adenopathy, arthritis and more than 30 arthralgia-involved joints. Both combinations have sensitivity and positive predictive value above 90%. The combination using arthralgia alone has better specificity (83%) than the broader combination (75%). Yet, the broader combination has a higher negative predictive value (Table 4).

### 3.4. Phylogenetic Analysis

We obtained ten complete *E1* gene sequences from patients’ isolates. Nucleotide identity among the sequences was 99.6–100% and 99.8–100% at the amino acid level. When compared to the British Virgin Island’s isolate (KJ451624.1), we found nine synonymous substitutions and one non-synonymous substitution. The non-synonymous substitution occurred in the E1 protein at the nucleotide position 3452 (C → T) changing the amino acid residue 342 (A → V).

**Figure 3:**
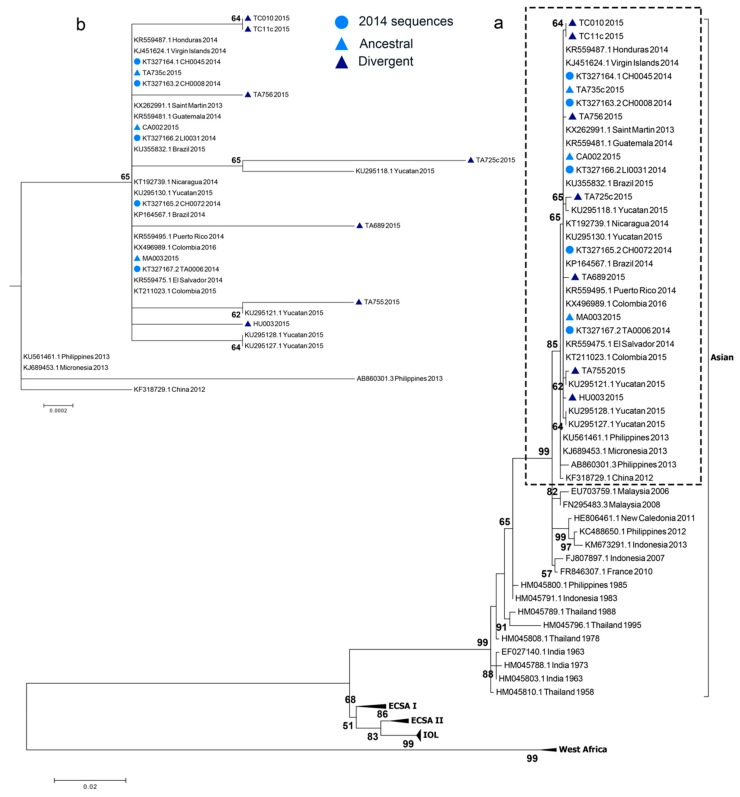
Maximum Likelihood phylogeny based on the *E1* gene of 73 CHIKV sequences. The four CHIKV lineages are shown. Bold numbers at nodes correspond to the bootstrap value. Only bootstrap values above 50 are shown. The tree is drawn to scale, with branch lengths measured in the number of substitutions per site. (**a**) Strains isolated from Tapachula, Chiapas, 2015 belong to the Asian lineage. (**b**) Magnification of the discontinue box in (**a**), that shows the relationship of the newly and previously [42] sequenced strains. Sequences that were identical to the previously reported were considered ancestral, the others were considered divergent. The previously sequenced strains are indicated with a circle. The newly sequenced strains are indicated with a triangle. The triangles of the divergent sequences are darker.

**Figure 4:**
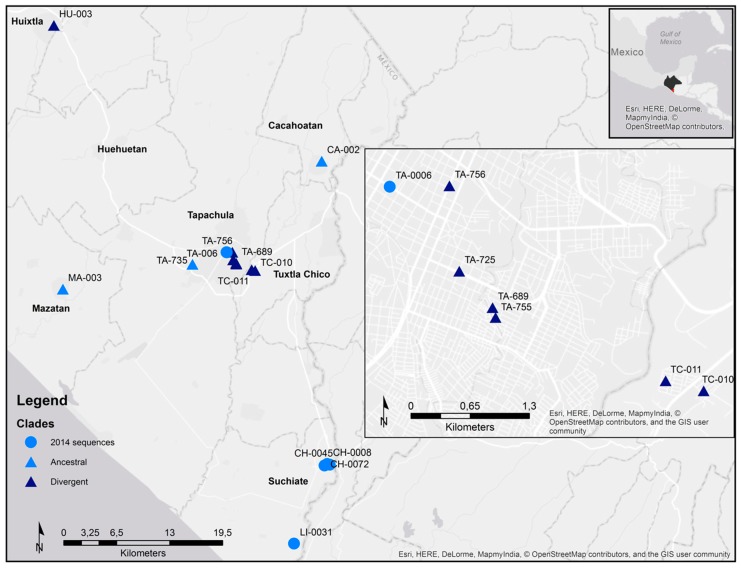
Chikungunya virus strains distribution across Chiapas, Mexico, 2014–2015. The different municipalities were CHIKV was isolated and sequenced are labeled. The upper right square indicates the location of Tapachula municipality and Chiapas state. The middle right square is a magnification of the sequences from Tapachula and Tuxtla Chico. The previously sequenced strains [42] are indicated with a circle. The newly sequenced strains are indicated with a triangle. The triangles of the divergent sequences are darker. We used the World Light Gray base by Esri (Esri Inc.) [41].

The maximum-likelihood and Bayesian approaches showed that the newly obtained CHIKV sequences clustered with the rest of the Caribbean isolates in the Asian lineage (Figure 3a). Former reported CHIKV sequences from Chiapas state in 2014 [42], clustered with the sequences reported from other countries in the Caribbean, Central and South America, from 2013 to 2016. Three of the newly obtained sequences clustered with the formerly reported sequences from Chiapas. These sequences were from Cacahoatan, Mazatan and Tapachula. Four of the sequences formed their own monophyletic group with a bootstrap support and posterior probability greater than 60 (Figure S1). One monophyletic group was composed of the two samples from Tuxtla Chico. The two remaining groups were made of a sample from Tapachula and a sample reported from Yucatán state. Two samples from Tapachula and one from Huixtla diverged from the former sequenced viruses but with low bootstrap and posterior probability values (Figure 3b). Sequences that were identical to the former reported Chiapas’ strains were denominated ancestral. Not identical samples were denominated divergent. There were several Chikungunya viruses circulating in Chiapas state during June and July 2015 (Figure 4). Different viruses were circulating in the city of Tapachula. It is interesting that patients from the same infection cluster were infected with different viruses. Such is the case of strains TA-689 and TA-755 (Figure 1 and Figure 3b). Even though patients had their dwellings separated 110 m from each other, the strains had three transitions and one transversion. Strain TA-755 grouped with a strain from Yucatan with a high bootstrap and posterior probability value, confirming the difference from strain TA-689.

### 3.5. Chikungunya Virus Strain—Specific Clinical Manifestations

We compared the clinical manifestations of the patients with sequenced CHIKV strains. The patients were grouped according to their infecting strain, ancestral or divergent. Three patients formed the ancestral group and seven patients the divergent group. The ten studied patients had a mean age of 44 years old and 60% were female. There was no difference regarding age and gender between the two groups.

Patients from both groups showed a similar symptomatology. Nevertheless, patients infected with the divergent strains reported arthritis and asthenia at a significantly greater proportion. Adenopathy and vertigo were also present in greater proportion, but not significant. Even though the ten patients reported gastrointestinal symptoms, only patients in the divergent group reported abdominal pain and diarrhea (Table 5). There was no difference in the arthralgia distribution. Patients from the divergent group reported arthritis mostly in the proximal interphalangeal joints and the ankle (both 43%). Patients from the ancestral group only reported rash at the face and neck in one patient. In contrast, two patients from the divergent group reported rash at the arms and forearms.

Sixty-seven percent of the patients in the divergent group needed help to fulfill their daily activities, compared to the ancestral group, which no one needed help. The most needed help was to lift from a sitting position in 67% of the patients. Patients from the divergent group reported a higher pain (median 9) compared to the ancestral group (median 7) but not significant.

We were able to obtain the sequence of the CHIKV strain that infected three of the four chronic cases (TC11, TA756 and TA755). The three sequences diverged from the previously reported E1 sequences. Two of them (TC11 and TA755) formed their own monophyletic group.

The patient that was infected with the strain TA725, the virus containing the non-synonymous mutation A342V, had certain clinical remarks. It was the patient with a higher number of arthritis-involved joints (metacarpophalangeal, proximal interphalangeal, distal interphalangeal, knee and ankle). It was one of the patients that reported a VAS score of 10. Also, it was one of the two patients that needed help to fulfill the four evaluated activities (walk, lift from a sitting position, eat and bathe).

## 4. Discussion

We were able to identify 52 patients with confirmed acute CHIKF from eight different municipalities in the state of Chiapas. In average we saw 4.7 CHIKV-confirmed patients per weekday. The patients who sought medical attention were middle-aged. This could be explained because the health clinic receives only State workers and their immediate relatives, not open to the general population. We found a higher proportion of affected females as reported by Danis-Lozano [28]. This could be due to CHIKV’s home environment transmission as described previously in dengue seroprevalence reports [43]. Also, a social explanation could be possible. Thompson reported that women visited their primary care provider to a greater extent than did men for both physical and mental health concerns [44].

We were not able to identify the etiological agent of the acute undifferentiated febrile illnesses. Possibly the viruses were cleared in the first days post-onset of symptoms, before patients sought medical attention. Another possibility is that patients started with mild symptomatology and didn’t consider themselves sick, arriving too late for viral RNA detection.

Several patients’ dwellings formed clusters, while others were isolated. The isolated patients can be explained by the fact that the patients’ neighbors probably were not State workers, therefore sought medical attention at another place.

When compared to the other studies from Mexico [26,27,28], arthralgia, headache, and shivers were present in the same proportion. Arthritis and adenopathy were only reported in patients from Chiapas. We don’t think that these manifestations are population or virus specific. As the obtained information from Garay-Moran’s study [27] came from a previously established database, arthritis could not have been considered even if it was manifested by the patients. Patients from Yucatan may have manifested arthritis but not taken in consideration. Patients infected with viruses from the ECSA, IOL and Asian lineages reported arthritis [8,18,22,23,45,46,47,48,49,50,51,52]. Adenopathy was previously reported in La Réunion [22,23], India [53,54] and Sri Lanka [55,56], where the Indian Ocean Lineage was circulating. In America, Colombia and Grenada reported patients with adenopathy [15,50]; as well as travelers from Spain that visited Dominican Republic or Haiti [57]. Just two studies mention the localization of the affected lymph nodes, with cervical being the most commonly affected [53,57]. In our study, we found retroauricular adenopathy, which can be seen as inflammation of the external ear. Javelle reported inflammation of the external ear in patients with acute Chikungunya infection at Malaysia. They suggest looking for inflammation of external ears in all suspected CHIKV cases [58]. Probably CHIKV can be disseminated from the retroauricular lymph nodes to the external ear, or vice versa.

We observed a greater proportion of patients with hemorrhagic signs than the rest of the Asian lineage-infected patients. This proportion difference is significant. We are not the only ones who reported a great proportion in hemorrhagic signs. Staikowsky reported hemorrhagic signs in 28% of the studied patients from La Réunion [22].

In this study, we report a greater proportion of arthralgia-involved joints. Arthralgia in shoulder, wrist, metacarpophalangeal joints, hip, knee and ankle were affected in a greater proportion than patients from La Réunion, India, Sri Lanka, Congo, and Gabon [22,46,55,59,60]. In contrast, cervical arthralgia was affected at a lower proportion. Arthralgia in elbow, proximal interphalangeal, and metatarsophalangeal joints was distributed in the same proportion as in other studies [8,23,59]. The patients studied manifested lower proportion of rash affected areas when compared to the available data from India and La Réunion [22,61]. Only rash on the arm, forearm, and thighs were affected in a similar proportion.

Patients with confirmed CHIKF complained of higher pain levels than patients with AUFI, as it was confirmed by the multivariable analysis. Women reported higher pain. This could be explained because women have greater pain sensitivity compared with men for most experimental pain modalities [62].

We are the first ones to report chronic post-chikungunya arthralgia in Mexican population after 14 months of onset of symptoms. Patients had an arthralgia-free period nevertheless it relapsed. Hand arthralgia resolved, but knee arthralgia persisted during this period of time. The proportion of the arthralgia-involved joints at 14 months differs from the reported by Schilte [7]. Fifty percent of the patients from Schilte’s study in La Réunion had hand, knee and ankle arthralgia after 14 months. However, Schilte studied 76 patients giving confidence to their study. In contrast, only four out of 52 of our patients could be interviewed by phone after 14 months of onset of symptoms. When we tried to contact the patients, the phone number was incorrect, never answered or refused the interview. This is a great bias; therefore, we included it for descriptive purposes and as evidence that Mexican patients can continue with arthralgia at least 14 months after onset of symptoms.

We investigated the possibility of using combinations of clinical features for differentiating between patients with CHIKF and patients with AUFI. The combination of arthralgia in wrist, metacarpophalangeal joints and knee has a better specificity. Combination of arthralgia in the same places plus adenopathy, arthritis and more than 30 arthralgia involved joints had a higher negative predictive value.

Furthermore, we sequenced the CHIKV infecting strains and associate the viruses with the different clinical presentation. We reconfirmed that the Asian lineage was affecting the state of Chiapas and was the responsible for the outbreak. We didn’t find the reported Brazilian ECSA CHIKV in Mexico. Also, we didn’t find the single adaptive mutation in *E1* (A226V) that confers a fitness advantage for IOL strains in *A. albopictus*.

Here we found different circulating CHIKV strains. This can be explained because samples were taken when the outbreak was still raging. Two of the sequences grouped with strains reported from Yucatán. The collection date of the Yucatan samples is unknown, so we can only infer that viruses were circulating between both states. It would be interesting to obtain the sequences of the viruses from the following years to determine which of the clades persisted through time. In addition, the whole genome sequencing could reveal a better picture of the virus phylogeny.

When the different CHIKV sequenced strains were associated with their spatial location in the infection clusters there was no coincidence. Samples TA-689 and TA-755 shared the infection cluster but were not in the same monophyletic group. It was not possible to sample every infection clusters because most of the clusters were composed of IgM+ patients, so no viral RNA could be isolated and sequenced.

We observed that patients infected with the divergent strain had more clinical features than patients infected with the ancestral clade. This needs to be investigated thoroughly. This is the first study to compare the clinical features of patients associated with CHIKV inter-lineage variation. The patient infected with the strain that contained the non-synonymous mutation, A342V, had several remarks. Even though both amino acids have a hydrophobic side chain, the effect in the viral protein and its interaction with the host yet has to be determined.

We are aware that our study has limitations and should be addressed in the future. We only obtained patients from one hospital that gives service to a specific type of population; therefore, our sample was not truly representative of the general population. Also, our number of chikungunya-negative patients was small to make strong associations and fully differentiate CHIKF effectively. We were not able to establish a specific etiological agent for the Chikungunya-negative patients. The number of patients infected with the ancestral clade is low, so findings are simply observational.

## 5. Conclusions

We defined the complete clinical features of Chikungunya fever in 52 confirmed patients from Southeastern Mexico. We obtained 10 new CHIKV E1 sequences and identified co-circulation of CHIKV strains in the state of Chiapas. Different CHIKV strains were associated with different clinical features. These findings can help to distinguish CHIKF from other febrile diseases and provide information about genomic variants of this virus in Mexico.

## Figures and Tables

**Table 1:** Clinical features of the studied patients in Tapachula, Chiapas, June through July 2015 ^a^.

Symptom	CHIKV+ *n* = 52	AUFI *n* = 9	*p*-Value ^b^	rRT-PCR *n* = 33	IgM *n* = 19	*p*-Value ^c^
Arthralgia	51 (98)	8 (89)	0.275	33 (100)	18 (95)	0.365
Myalgia	51 (98)	9 (100)	1.000	33 (100)	18 (95)	0.365
Headache	47 (90)	8 (89)	1.000	32 (97)	15 (79)	0.054
Retroocular pain	16 (31)	4 (44)	0.458	12 (36)	4 (21)	0.353
Conjunctival hyperemia	21 (40)	3 (33)	1.000	13 (39)	8 (42)	1.000
Shivers	44 (85)	6 (67)	0.343	29 (88)	15 (79)	0.443
Asthenia	39 (75)	4 (44)	0.108	25 (76)	14 (74)	1.000
Rash	32 (62)	5 (56)	0.729	18 (55)	14 (74)	0.240
Pruritus	35 (67)	5 (56)	0.706	22 (67)	13 (68)	1.000
Arthritis *	34 (65)	1 (11)	0.003 *	21 (64)	13 (68)	0.771
Adenopathy	25 (48)	1 (11)	0.065	13 (39)	12 (63)	0.150
Vertigo	17 (33)	3 (33)	1.000	9 (27)	8 (42)	0.360
Gastrointestinal symptoms	50 (96)	8 (89)	0.386	32 (97)	18 (95)	1.000
Abdominal pain	20 (39)	3 (33)	1.000	13 (39)	7 (37)	1.000
Anorexia	40 (77)	6 (67)	0.676	27 (82)	13 (68)	0.317
Dysgeusia	39 (75)	4 (44)	0.108	26 (79)	13 (68)	0.510
Diarrhea	15 (29)	5 (56)	0.139	7 (21)	8 (42)	0.126
Nausea	34 (65)	5 (56)	0.710	24 (73)	10 (53)	0.226
Vomit	5 (10)	2 (22)	0.273	2 (6)	3 (16)	0.342
Hemorrhagic signs	12 (23)	4 (44)	0.224	7 (21)	5 (26)	0.739
Epistaxis	3 (6)	0	1.000	1 (3)	2 (11)	0.546
Gingivorrhagia	5 (10)	2 (22)	0.273	4 (12)	1 (5)	0.641
Petechiae	5 (10)	2 (22)	0.273	3 (9)	2 (11)	1.000

^a^ Values are no. (%) patients. CHIKV, chikungunya virus; AUFI, acute undifferentiated fever illness; rRT-PCR, real-time reverse transcription PCR; IgM: immunoglobulin M. ^b^ CHIKV+ vs. AUFI. ^c^ rRT-PCR vs. IgM. * *p* < 0.01. Fisher exact test.

**Table 2:** Arthritis and Rash distribution of the studied patients in Tapachula, Chiapas, June through July 2015 ^a^.

Symptoms		CHIKV+ *n* = 52	AUFI *n* = 9	*p*-Value ^b^	rRT-PCR *n* = 33	IgM *n* = 19	*p*-Value ^c^
Arthritis	Wrist	1 (2)	0	1.000	1 (3)	0	1.000
	MCP	2 (4)	0	1.000	2 (6)	0	0.527
	PIP	4 (8)	0	1.000	4 (12)	0	0.284
	DIP	2 (3)	0	1.000	2 (6)	0	0.527
	Knee	5 (10)	0	1.000	4 (12)	1 (5)	1.000
	Ankle	13 (25)	1 (11)	0.430	9 (27)	4 (21)	1.000
	MTP	1 (2)	0	1.000	1 (3)	0	1.000
Rash	Face	1 (2)	0	1.000	1 (3)	0	1.000
	Neck	2 (4)	1 (11)	0.386	1 (3)	1 (5)	1.000
	Chest	7 (14)	3 (33)	0.157	3 (9)	4 (21)	0.400
	Abdomen	10 (19)	1 (11)	1.000	4 (12)	6 (32)	0.142
	Forearm	18 (35)	4 (44)	0.710	9 (27)	9 (47)	0.226
	Arm	16 (31)	3 (33)	1.000	8 (24)	8 (42)	0.220
	Thigh	12 (23)	1 (11)	0.669	6 (18)	6 (32)	0.317
	Leg	9 (17)	0	0.332	3 (9)	6 (32)	0.059
	Feet	1 (2)	0	1.000	0	1 (5)	0.365
	Back	9 (17)	0	0.361	4 (12)	5 (26)	0.260
	Buttocks	4 (8)	0	1.000	1 (3)	3 (16)	0.132

^a^ Values are no. (%) patients. CHIKV, chikungunya virus; MCP, metacarpophalangeal joint; PIP, proximal interphalangeal joint; DIP, distal interphalangeal joint; MTP, metatarsophalangeal joint. ^b^ CHIKV+ vs. AUFI. ^c^ rRT-PCR vs. IgM.

**Table 3:** Multivariate analysis associated with Chikungunya fever in southeastern Mexican population ^a^.

Parameter	Adjusted Odds Ratio ^b^	(95% Confidence Interval)	*p*-Value
Arthritis			
Yes	6.61	1.24–35.19	0.027
Wrist arthralgia			
Yes	22.1	2.58–188.74	0.005
MCP arthralgia			
Yes	6.2	1.25–30.67	0.025
Knee arthralgia			
Yes	5.89	1.17–29.49	0.031
VAS score	1.61	1.066–2.440	0.024
Number of involved joints	1.05	1.004–1.100	0.033

^a^ MCP, metacarpophalangeal; VAS, visual analog scale. ^b^ Adjusted for age and gender.

**Table 4:** Clinical features used to distinguish patients with Chikungunya fever from patients with AUFI ^a^.

Parameter	PPV (%)	NPV (%)	Sens (%)	Spec (%)	Likelihood Ratio (+)	Likelihood Ratio (−)
Wrist arthralgia	96.1	44.4	90	66.6	2.72	0.13
Wrist, MCP and knee arthralgia	98	55	92.7	83.3	5.56	0.08
Wrist, MCP and knee arthralgia, plus arthritis (or adenopathy)	98	44.4	91	80	4.55	0.11
MCP and knee arthralgia plus ≥8 VAS score	96	62.5	94.2	71.4	3.29	0.08
Wrist, MCP and knee arthralgia, ≥30 joints involved, arthritis and adenopathy	96.1	66.6	94.3	75	3.77	0.07

^a^ AUFI, acute undifferentiated febrile illness; PPV, positive predictive value; NPV, negative predictive value; Sens, sensitivity; Spec, specificity; MCP, metacarpophalangeal; VAS, visual analog scale.

**Table 5:** Clinical features of patients infected with the isolated CHIKV strains ^a^.

Symptoms	Ancestral *n* = 3	Divergent *n* = 7	*p*-Value
Arthralgia	3 (100)	7 (100)	^b^
Myalgia	3 (100)	7 (100)	^b^
Headache	3 (100)	6 (86)	1.000
Retroocular pain	1 (33)	1 (14)	1.000
Conjunctival hyperemia	3 (100)	3 (43)	0.200
Shivers	3 (100)	7 (100)	^b^
Asthenia *	0	6 (86)	0.033 *
Rash	1 (33)	2 (29)	1.000
Pruritus	1 (33)	5 (71)	0.500
Arthritis *	0	6 (86)	0.033 *
Adenopathy	0	4 (57)	0.200
Vertigo	0	2 (27)	1.000
Gastrointestinal symptoms	3 (100)	7 (100)	^b^
Abdominal pain	0	3 (43)	0.475
Anorexia	2 (67)	6 (86)	1.000
Dysgeusia	2 (67)	6 (86)	1.000
Diarrhea	0	2 (29)	1.000
Nausea	2 (67)	5 (71)	1.000
Hemorrhagic signs	0	1 (14)	1.000
Epistaxis	0	1 (14)	1.000

^a^ Values are no. (%) patients. ^b^ No statistics were computed because the variable is a constant. CHIKV, Chikungunya virus. * *p* < 0.01. Fisher exact test.

## Supplementary Materials

The following are available online at http://www.mdpi.com/1999-4915/10/5/248/s1, Table S1: Chikungunya virus strains used in this study; Figure S1: Maximum clade credibility (MCC) phylogeny based on the *E1* gene of 73 CHIKV sequences.

## References

[1] Weaver S.C., Lecuit M. (2015). Chikungunya Virus and the Global Spread of a Mosquito-Borne Disease. N. Engl. J. Med..

[2] Simon F., Javelle E., Oliver M., Leparc-Goffart I., Marimoutou C. (2011). Chikungunya virus infection. Curr. Infect. Dis. Rep..

[3] Robinson M.C. (1955). An epidemic of virus disease in Southern Province, Tanganyika Territory, in 1952–1953. I. Clinical features. Trans. R. Soc. Trop. Med. Hyg..

[4] Caglioti C., Lalle E., Castilletti C., Carletti F., Capobianchi M.R., Bordi L. (2013). Chikungunya virus infection: An overview. New Microbiol..

[5] Thiberville S.D., Boisson V., Gaudart J., Simon F., Flahault A., de Lamballerie X. (2013). Chikungunya fever: A clinical and virological investigation of outpatients on Reunion Island, South-West Indian Ocean. PLoS Negl. Trop. Dis..

[6] Pialoux G., Gaüzère B.-A., Jauréguiberry S., Strobel M. (2007). Chikungunya, an epidemic arbovirosis. Lancet Infect. Dis..

[7] Schilte C., Staikowsky F., Staikovsky F., Couderc T., Madec Y., Carpentier F., Kassab S., Albert M.L., Lecuit M., Michault A. (2013). Chikungunya virus-associated long-term arthralgia: A 36-month prospective longitudinal study. PLoS Negl. Trop. Dis..

[8] Borgherini G., Poubeau P., Staikowsky F., Lory M., Le Moullec N., Becquart J.P., Wengling C., Michault A., Paganin F. (2007). Outbreak of chikungunya on Reunion Island: Early clinical and laboratory features in 157 adult patients. Clin. Infect. Dis..

[9] Pan American Health Organization Chikungunya: Data, Maps and Statistics. http://www.paho.org/hq/index.php?option=com_topics&view=readall&cid=5927&Itemid=40931&lang=en.

[10] Dirección General de Epidemiología Boletín Epidemiológico—Semana 44, 2017. https://www.gob.mx/cms/uploads/attachment/file/272138/sem44.pdf.

[11] Schwartz O., Albert M.L. (2010). Biology and pathogenesis of chikungunya virus. Nat. Rev. Microbiol..

[12] Schuffenecker I., Iteman I., Michault A., Murri S., Frangeul L., Vaney M.-C., Lavenir R., Pardigon N., Reynes J.-M., Pettinelli F. (2006). Genome Microevolution of Chikungunya Viruses Causing the Indian Ocean Outbreak. PLoS Med..

[13] De Lamballerie X., Leroy E., Charrel R.N., Ttsetsarkin K., Higgs S., Gould E.A. (2008). Chikungunya virus adapts to tiger mosquito via evolutionary convergence: A sign of things to come?. Virol. J..

[14] Sahadeo N., Mohammed H., Allicock O.M., Auguste A.J., Widen S.G., Badal K., Pulchan K., Foster J.E., Weaver S.C., Carrington C.V.F. (2015). Molecular Characterisation of Chikungunya Virus Infections in Trinidad and Comparison of Clinical and Laboratory Features with Dengue and Other Acute Febrile Cases. PLoS Negl. Trop. Dis..

[15] Mattar S., Miranda J., Pinzon H., Tique V., Bolaños A., Aponte J., Arrieta G., Gonzalez M., Barrios K., Contreras H. (2015). Outbreak of chikungunya virus in the north caribbean area of colombia: Clinical presentation and phylogenetic analysis. J. Infect. Dev. Ctries..

[16] Chen R., Puri V., Fedorova N., Lin D., Hari K.L., Jain R., Rodas J.D., Das S.R., Shabman R.S., Weaver S.C. (2016). Comprehensive Genome-Scale Phylogenetic Study Provides New Insights on the Global Expansion of Chikungunya Virus. J. Virol..

[17] Nunes M.R.T., Faria N.R., de Vasconcelos J.M., Golding N., Kraemer M.U., de Oliveira L.F., da Silva Azevedo R.d.S., da Silva D.E.A., da Silva E.V.P., da Silva S.P. (2015). Emergence and potential for spread of Chikungunya virus in Brazil. BMC Med..

[18] Souza T.M.A., Azeredo E.L., Badolato-Corrêa J., Damasco P.V., Santos C., Petitinga-Paiva F., Nunes P.C.G., Barbosa L.S., Cipitelli M.C., Chouin-Carneiro T. (2017). First Report of the East-Central South African Genotype of Chikungunya Virus in Rio de Janeiro, Brazil. PLoS Curr..

[19] Dos Passos Cunha M., Dos Santos C.A., de Lima Neto D.F., Schanoski A.S., Pour S.Z., Passos S.D., DE Souza M.S.F., Costa D.D., de Andrade Zanotto P.M. (2017). Outbreak of chikungunya virus in a vulnerable population of Sergipe, Brazil—A molecular and serological survey. J. Clin. Virol..

[20] Leparc-Goffart I., Nougairede A., Cassadou S., Prat C., de Lamballerie X. (2014). Chikungunya in the Americas. Lancet.

[21] Burt F.J., Chen W., Miner J.J., Lenschow D.J., Merits A., Schnettler E., Kohl A., Rudd P.A., Taylor A., Herrero L.J. (2017). Chikungunya virus: An update on the biology and pathogenesis of this emerging pathogen. Lancet Infect. Dis..

[22] Staikowsky F., Le Roux K., Schuffenecker I., Laurent P., Grivard P., Develay A., Michault A. (2008). Retrospective survey of Chikungunya disease in Réunion Island hospital staff. Epidemiol. Infect..

[23] Staikowsky F., Talarmin F., Grivard P., Souab A., Schuffenecker I., Le Roux K., Lecuit M., Michault A. (2009). Prospective study of Chikungunya virus acute infection in the Island of La Reunion during the 2005–2006 outbreak. PLoS ONE.

[24] Yactayo S., Staples J.E., Millot V., Cibrelus L., Ramon-Pardo P. (2016). Epidemiology of Chikungunya in the Americas. J. Infect. Dis..

[25] Ramon-Pardo P., Cibrelus L., Yactayo S., Chikungunya Expert Group (2015). Chikungunya: Case definitions for acute, atypical and chronic cases. Conclusions of an expert consultation, Managua, Nicaragua, 20–21 May 2015. Wkly. Epidemiol. Rec..

[26] Cigarroa-Toledo N., Blitvich B.J., Cetina-Trejo R.C., Talavera-Aguilar L.G., Baak-Baak C.M., Torres-Chablé O.M., Hamid M.-N., Friedberg I., González-Martinez P., Alonzo-Salomon G. (2016). Chikungunya Virus in Febrile Humans and *Aedes aegypti* Mosquitoes, Yucatan, Mexico. Emerg. Infect. Dis..

[27] Garay-Morán C., Román-Pedroza J.F., López-Martínez I., Rodríguez-Martínez J.C., Ruiz-Matus C., Kuri-Morales P., Díaz-Quiñonez J.A. (2017). Clinical and epidemiological characterization of chikungunya fever in Mexico. Rev. Panam. Salud Publica.

[28] Danis-Lozano R., Díaz-González E.E., Trujillo-Murillo K.D.C., Caballero-Sosa S., Sepúlveda-Delgado J., Malo-García I.R., Canseco-Ávila L.M., Salgado-Corsantes L.M., Domínguez-Arrevillaga S., Torres-Zapata R. (2017). Clinical characterization of acute and convalescent illness of confirmed chikungunya cases from Chiapas, S. Mexico: A cross sectional study. PLoS ONE.

[29] World Health Organization The Mosquito. http://www.who.int/denguecontrol/mosquito/en/.

[30] Lanciotti R.S., Kosoy O.L., Laven J.J., Panella A.J., Velez J.O., Lambert A.J., Campbell G.L. (2007). Chikungunya virus in US travelers returning from India, 2006. Emerg. Infect. Dis..

[31] Seah C.L., Chow V.T., Chan Y.C. (1995). Semi-nested PCR using NS3 primers for the detection and typing of dengue viruses in clinical serum specimens. Clin. Diagn. Virol..

[32] Lanciotti R.S., Kosoy O.L., Laven J.J., Velez J.O., Lambert A.J., Johnson A.J., Stanfield S.M., Duffy M.R. (2008). Genetic and serologic properties of Zika virus associated with an epidemic, Yap State, Micronesia, 2007. Emerg. Infect. Dis..

[33] Erasmus J.H., Needham J., Raychaudhuri S., Diamond M.S., Beasley D.W.C., Morkowski S., Salje H., Fernandez Salas I., Kim D.Y., Frolov I. (2015). Utilization of an Eilat Virus-Based Chimera for Serological Detection of Chikungunya Infection. PLoS Negl. Trop. Dis..

[34] Morales-González K.R., Zomosa-Signoret V., Rivas-Estilla A.M., Vidaltamayo R. Improving Specificity of DENV E Proteins through Synthetic Sequence Redesign.

[35] Tamura K., Stecher G., Peterson D., Filipski A., Kumar S. (2013). MEGA6: Molecular Evolutionary Genetics Analysis version 6.0. Mol. Biol. Evol..

[36] Drummond A.J., Suchard M.A., Xie D., Rambaut A. (2012). Bayesian phylogenetics with BEAUti and the BEAST 1.7. Mol. Biol. Evol..

[37] Hasegawa M., Kishino H., Yano T. (1985). Dating of the human-ape splitting by a molecular clock of mitochondrial DNA. J. Mol. Evol..

[38] Rambaut A., Drummond A.J., Xie D., Baele G., Suchard M.A. Tracer v1.6. http://beast.community/tracer.

[39] Trpis M., Hausermann W. (1986). Dispersal and other population parameters of *Aedes aegypti* in an African village and their possible significance in epidemiology of vector-borne diseases. Am. J. Trop. Med. Hyg..

[40] Honório N.A., Silva W.D.C., Leite P.J., Gonçalves J.M., Lounibos L.P., Lourenço-de-Oliveira R. (2003). Dispersal of *Aedes aegypti* and *Aedes albopictus* (Diptera: Culicidae) in an urban endemic dengue area in the State of Rio de Janeiro, Brazil. Mem. Inst. Oswaldo Cruz.

[41] Esri Inc.; World Light Gray Base. http://www.arcgis.com/home/item.html?id=ed712cb1db3e4bae9e85329040fb9a49.

[42] Kautz T.F., Díaz-González E.E., Erasmus J.H., Malo-García I.R., Langsjoen R.M., Patterson E.I., Auguste D.I., Forrester N.L., Sanchez-Casas R.M., Hernández-Ávila M. (2015). Chikungunya Virus as Cause of Febrile Illness Outbreak, Chiapas, Mexico, 2014. Emerg. Infect. Dis..

[43] Ramos-Castañeda J., Sepúlveda-Salinas K.J., Mayer S.V., Falcón-Lezama J.A., Galeana-Hernández M., Amaya-Larios I.Y., Vasilakis N., Comas-García A., Martínez-Vega R.A. (2014). Seroprevalence of Neutralizing Antibodies Against Dengue Virus in Two Localities in the State of Morelos, Mexico. Am. J. Trop. Med. Hyg..

[44] Thompson A.E., Anisimowicz Y., Miedema B., Hogg W., Wodchis W.P., Aubrey-Bassler K. (2016). The influence of gender and other patient characteristics on health care-seeking behaviour: A QUALICOPC study. BMC Fam. Pract..

[45] Lakshmi V., Neeraja M., Subbalaxmi M.V., Parida M.M., Dash P.K., Santhosh S.R., Rao P.V.L. (2008). Clinical features and molecular diagnosis of Chikungunya fever from South India. Clin. Infect. Dis..

[46] Anish T.N., George B., Lawrence T., Muthukkutty S., Ramachandran R., Vijayakumar K. (2011). Clinical Profile of Chikungunya Patients during the Epidemic of 2007 in Kerala, India. J. Glob. Infect. Dis..

[47] Kularatne S.A.M., Weerasinghe S.C., Gihan C., Wickramasinghe S., Dharmarathne S., Abeyrathna A., Jayalath T. (2012). Epidemiology, clinical manifestations, and long-term outcomes of a major outbreak of Chikungunya in a Hamlet in Sri Lanka, in 2007: A longitudinal cohort study. J. Trop. Med..

[48] Dutta S.K., Pal T., Saha B., Mandal S., Tripathi A. (2014). Copy number variation of chikungunya ECSA virus with disease symptoms among Indian patients. J. Med. Virol..

[49] Rowhani-Rahbar A., Ellis E.M., Feldstein L.R., Ellis B.R., Halloran M.E. (2016). The First Reported Outbreak of Chikungunya in the U.S. Virgin Islands, 2014–2015. Am. J. Trop. Med. Hyg..

[50] Macpherson C., Noël T., Fields P., Jungkind D., Yearwood K., Simmons M., Widjaja S., Mitchell G., Noel D., Bidaisee S. (2016). Clinical and Serological Insights from the Asian Lineage Chikungunya Outbreak in Grenada, 2014: An Observational Study. Am. J. Trop. Med. Hyg..

[51] Tomashek K.M., Lorenzi O.D., Andújar-Pérez D.A., Torres-Velásquez B.C., Hunsperger E.A., Munoz-Jordan J.L., Perez-Padilla J., Rivera A., Gonzalez-Zeno G.E., Sharp T.M. (2017). Clinical and epidemiologic characteristics of dengue and other etiologic agents among patients with acute febrile illness, Puerto Rico, 2012–2015. PLoS Negl. Trop. Dis..

[52] Zingman M.A., Paulino A.T., Payano M.P. (2017). Clinical manifestations of chikungunya among university professors and staff in Santo Domingo, the Dominican Republic. Rev. Panam. Salud Publica.

[53] Suryawanshi S.D., Dube A.H., Khadse R.K., Jalgaonkar S.V., Sathe P.S., Zawar S.D., Holay M.P. (2009). Clinical profile of chikungunya fever in patients in a tertiary care centre in Maharashtra, India. Indian J. Med. Res..

[54] Keny M., Pereira I., deSa S., Gomes E. (2014). Painful cervical lymphadenopathy: An unusual presentation of chikungunya. Int. J. Appl. Basic Med. Res..

[55] Kularatne S.A.M., Gihan M.C., Weerasinghe S.C., Gunasena S. (2009). Concurrent outbreaks of Chikungunya and Dengue fever in Kandy, Sri Lanka, 2006–2007: A comparative analysis of clinical and laboratory features. Postgrad. Med. J..

[56] Reller M.E., Akoroda U., Nagahawatte A., Devasiri V., Kodikaarachchi W., Strouse J.J., Chua R., Hou Y., Chow A., Sessions O.M. (2013). Chikungunya as a Cause of Acute Febrile Illness in Southern Sri Lanka. PLoS ONE.

[57] Norman F.F., Monge-Maillo B., Perez-Molina J.-A., de Ory F., Franco L., Sánchez-Seco M.-P., López-Vélez R. (2015). Lymphadenopathy in Patients with Chikungunya Virus Infection Imported from Hispaniola: Case Reports. J. Travel Med..

[58] Javelle E., Tiong T.H., Leparc-Goffart I., Savini H., Simon F. (2014). Inflammation of the external ear in acute chikungunya infection: Experience from the outbreak in Johor Bahru, Malaysia, 2008. J. Clin. Virol..

[59] Moyen N., Thiberville S.-D., Pastorino B., Nougairede A., Thirion L., Mombouli J.-V., Dimi Y., Leparc-Goffart I., Capobianchi M.R., Lepfoundzou A.D. (2014). First reported chikungunya fever outbreak in the republic of Congo, 2011. PLoS ONE.

[60] Caron M., Paupy C., Grard G., Becquart P., Mombo I., Nso B.B.B., Kassa Kassa F., Nkoghe D., Leroy E.M. (2012). Recent Introduction and Rapid Dissemination of Chikungunya Virus and Dengue Virus Serotype 2 Associated With Human and Mosquito Coinfections in Gabon, Central Africa. Clin. Infect. Dis..

[61] Kawle A.P., Nayak A.R., Bhullar S.S., Borkar S.R., Patankar S.D., Daginawala H.F., Singh L.R., Kashyap R.S. (2017). Seroprevalence and clinical manifestations of chikungunya virus infection in rural areas of Chandrapur, Maharashtra, India. J. Vector Borne Dis..

[62] Fillingim R.B., King C.D., Ribeiro-Dasilva M.C., Rahim-Williams B., Riley J.L. (2009). Sex, gender, and pain: A review of recent clinical and experimental findings. J. Pain.

