# Supplementary material for: Molecular and Clinical Characterization of Chikungunya Virus Infections in Southeast Mexico
Source: Viruses. 2018 May 9;10(5):248. doi: 10.3390/v10050248 (PMC5977241; doi:10.3390/v10050248)
Supplement: Supplementary file 1 [file viruses-10-00248-s001.pdf]

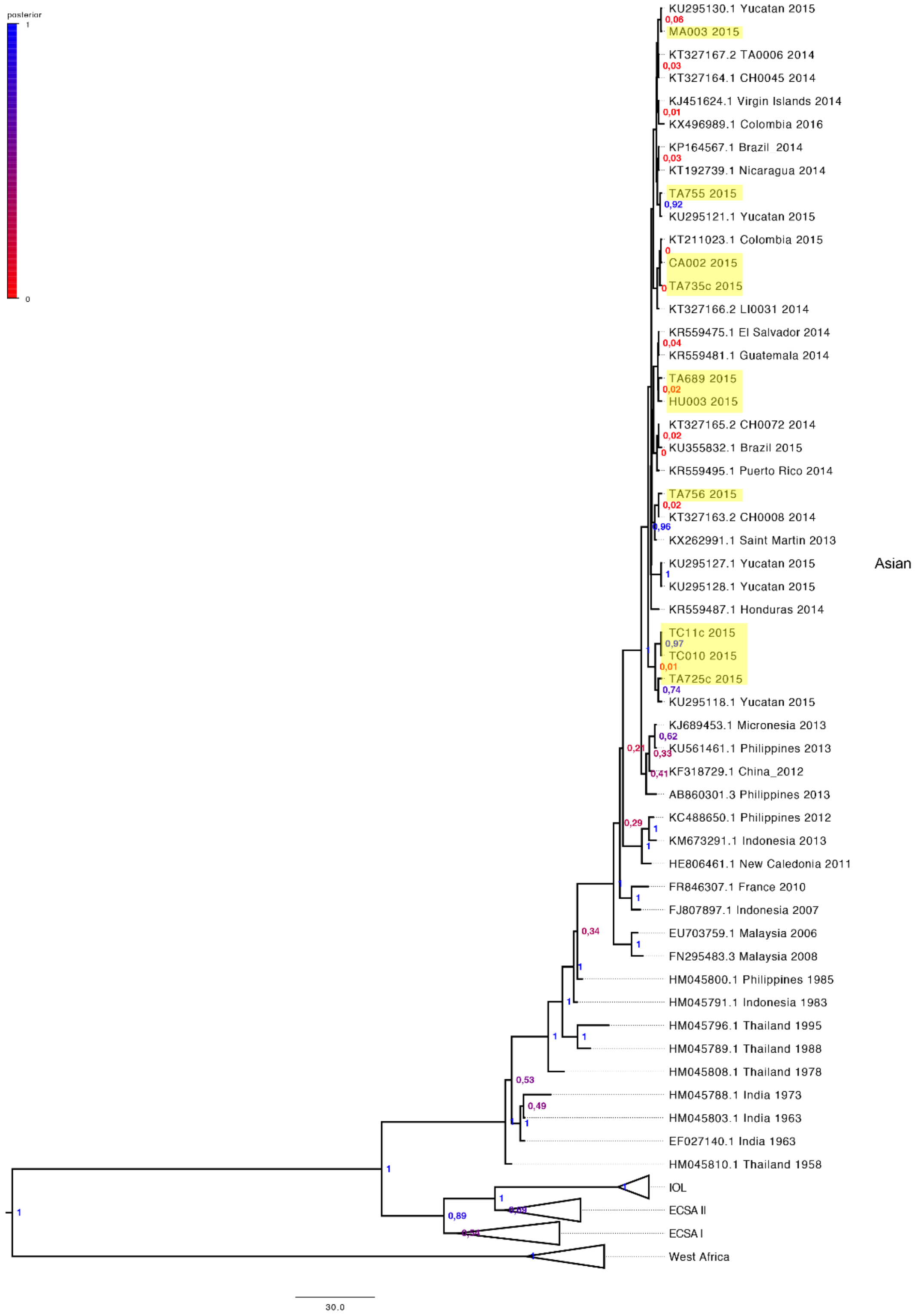

Figure S1. Maximum clade credibility (MCC) phylogeny based on the E1 gene of 73 CHIKV sequences. The four CHIKV lineages are shown. Sequences obtained from this study are highlighted in yellow. Nodes correspond to posterior probabilities.

Table S1. Chikungunya virus strains used in this study

Name	Accession number	Strain name	Year of isolation	State/Country of isolation	Lineage
Philippines 2013	AB860301.3	CHIKV-13-112A	2013	Philippines	Asian
Tanzania 1953	AF369024.2	S-27 African prototype	1953	Tanzania	ECSA I
Senegal 1983	AY726732.1	37997	1983	Senegal	West Africa
La Reunion 2006	DQ443544.2	LR2006_OPY1	2006	La Réunion	IOL
India 1963	EF027140.1	IND-63-WB1	1963	India	Asian
Italy 2007	EU244823.2	ITA07-RA1	2007	Italy	IOL
Malaysia 2006	EU703759.1	MY002IMR/06/BP	2006	Malaysia	Asian
Singapore 2008	FJ445510.2	SGEHICHS277108	2008	Singapore	IOL
Malaysia 2009	FJ807895.1	0901aTw	2009	Malaysia	IOL
Indonesia 2007	FJ807897.1	0706aTw	2007	Indonesia	Asian
Malaysia 2008	FN295483.3	MY/06/37348	2008	Malaysia	Asian
France 2010	FR846307.1	Pt11352	2010	France	Asian
China 2008	GU199351.1	SD08Pan	2008	China	IOL
Thailand 2009	GU301781.1	CHU-Chik683	2009	Thailand	IOL
New Caledonia 2011	HE806461.1	NC/2011-568	2011	New Caledonia	Asian
C.A.R 1984	HM045784.1	DakAR B 16878	1984	Central African Republic	ECSA II
India 1973	HM045788.1	PO731460	1973	India	Asian
Thailand 1988	HM045789.1	6441-88	1988	Thailand	Asian
Indonesia 1983	HM045791.1	JKT23574	1983	Indonesia	Asian
South Africa 1956	HM045792.1	Vereeniging	1956	South Africa	ECSA I
Thailand 1995	HM045796.1	CO392-95	1995	Thailand	Asian
Philippines 1985	HM045800.1	Hu/85/NR001	1985	Philippines	Asian

Sri Lanka 2007	HM045801.1	SL-CK1	2007	Sri Lanka	IOL
India 1963	HM045803.1	I-634029	1963	India	Asian
South Africa 1976	HM045805.1	AR 18211	1976	South Africa	ECSA I
Nigeria 1965	HM045807.1	IbAn4824	1965	Nigeria	West Africa
Thailand 1978	HM045808.1	3412-78	1978	Thailand	Asian
Congo 1960	HM045809.1	LSFS	1960	Congo	ECSA I
Thailand 1958	HM045810.1	TH35	1958	Thailand	Asian
Uganda 1982	HM045812.1	UgAg4155	1982	Uganda	ECSA II
Ivory Coast 1993	HM045820.1	ArA 30548	1993	Ivory Coast	West Africa
C.A.R. 1978	HM045822.1	HB78	1978	Central African Republic	ECSA II
Angola 1962	HM045823.1	Angola M2022	1962	Angola	ECSA II
India 2010	HM159387.1	TN06210	2010	India	IOL
Kenya 2004	HQ456255.1	Lamu33	2004	Kenya	IOL
India 2006	JF274082.1	IND-06-Guj	2016	India	IOL
Philippines 2012	KC488650.1	CHIKV-JC2012	2012	Philippines	Asian
Indonesia 2010	KC862329.1	NL10/152	2010	Indonesia	IOL
China 2012	KF318729.1	chik-sy	2012	China	Asian
Virgin Islands 2014	KJ451624.1	99659	2014	Virgin Islands	Asian
Micronesia 2013	KJ689453.1	Yap 13-2148	2013	Micronesia	Asian
Indonesia 2013	KM673291.1	DH130003	2013	Indonesia	Asian
Brazil 2014	KP164567.1	AMA2798/H804298	2014	Brazil	Asian
El Salvador 2014	KR559475.1	WHCHK6	2014	El Salvador	Asian
Guatemala 2014	KR559481.1	WHCHK12	2014	Guatemala	Asian
Honduras 2014	KR559487.1	WHCHK18	2014	Honduras	Asian
Puerto Rico 2014	KR559495.1	WHCHK26	2014	Puerto Rico	Asian

Nicaragua 2014	KT192739.1	7050	2014	Nicaragua	Asian
Colombia 2015	KT211023.1	440191	2015	Colombia	Asian
CH0008 2014	KT327163.2	CH0008	2014	Chiapas/Mexico	Asian
CH0045 2014	KT327164.1	CH0045	2014	Chiapas/Mexico	Asian
CH0072 2014	KT327165.2	CH0072	2014	Chiapas/Mexico	Asian
LI0031 2014	KT327166.2	LI0031	2014	Chiapas/Mexico	Asian
TA0006	KT327167.2	TA0006	2014	Chiapas/Mexico	Asian
Yucatan 2015	KU295118.1	YUC-M16	2015	Yucatan/Mexico	Asian
Yucatan 2015	KU295121.1	YUC-A36	2015	Yucatan/Mexico	Asian
Yucatan 2015	KU295127.1	YUC-A30	2015	Yucatan/Mexico	Asian
Yucatan 2015	KU295128.1	YUC-A15	2015	Yucatan/Mexico	Asian
Yucatan 2015	KU295130.1	YUC-A7	2015	Yucatan/Mexico	Asian
Brazil 2015	KU355832.1	RJ/CHIKV/2015	2015	Brazil	Asian
Philippines 2013	KU561461.1	1308aTw	2013	Philippines	Asian
Saint Martin 2013	KX262991.1	CHIKV/Homo sapiens/SXM/H-20235-STMARTIN-2013/2003	2013	Saint Martin	Asian
Colombia 2016	KX496989.1	Homo sapiens/COL/UF-1/2016	2016	Colombia	Asian

ECSA I, East Central South Africa I; ECSA II, East Central South Africa II; IOL, Indian Ocean lineage.
